# 植物源生物炭材料的制备及其在农药残留领域中的应用进展

**DOI:** 10.3724/SP.J.1123.2021.10024

**Published:** 2022-06-08

**Authors:** Xianzhao ZHANG, Dawei ZHEN, Fengmao LIU, Qingrong PENG, Zongyi WANG

**Affiliations:** 1.中国农业大学理学院应用化学系, 农药创新中心, 北京 100193; 1. Innovation Center of Pesticide Research, Department of Applied Chemistry, College of Science, China Agricultural University, Beijing 100193, China; 2.北京农学院, 农产品有害微生物及农残安全检测与控制北京市重点实验室, 北京 102206; 2. Beijing Key Laboratory of Detection and Control of Spoilage Microorganisms and Pesticide Residues in Agricultural Products, Beijing University of Agriculture, Beijing 102206, China

**Keywords:** 制备方法, 农药去除, 残留分析, 理论计算, 植物源生物炭材料, 综述, preparation method, pesticide removal, residues analysis, theoretical calculation, plant biomass-derived biochar, review

## Abstract

随着农药的广泛使用,其已普遍存在于环境中,对人们的身体健康产生巨大影响。因此,环境中农药残留的去除和分析检测对保护人体安全健康至关重要。同时,农药在环境中残留浓度低,需要一种对目标物有较强选择性和富集作用,并对环境影响小的前处理吸附剂。植物源生物炭是由植物源生物质作为碳源衍生得到的材料,其比表面积大、孔容量高、表面官能团可调节,且环境相容性好,其原料植物源生物质的价格低廉、来源广泛并可再生,是一种廉价高效的吸附剂。该文主要综述了近10年来植物源生物炭用于环境中农药残留去除和分析检测前处理的应用进展。其中在农药残留去除方面的应用主要包括降低农药在土壤中的移动性,修复手性农药造成的污染,负载降解农药的细菌及作为化肥的缓释载体。在农药残留分析检测前处理方面,植物源生物炭可用作分散固相萃取、固相微萃取和磁性固相萃取的吸附剂来选择性吸附水果和蔬菜中的有机磷类和三唑类农药,以及水环境中的有机氯类农药。另外,还介绍了植物源生物炭的吸附机理,详细阐述了基于计算模拟如密度泛函理论、分子动力学模拟和巨正则蒙特卡洛模拟的吸附机理研究并讨论了其优势。最后,总结了植物源生物炭在农药去除和农药残留分析检测前处理方面应用的优势,指出了其在农药残留领域应用待解决的问题。

## Abstract (en)

Pesticides such as insecticides, fungicides, and herbicides play an important role in the global agricultural industry as they reduce the occurrence of crop diseases, kill pests, and remove weeds. On the other hand, these pesticides are a double-edged sword because they have both acute effects and chronic adverse effects on human health. The widespread use of pesticides has led to their persistence in soil, water, and agricultural products, thus posing a serious threat to public health. Therefore, the removal and analysis of pesticides are critical to protecting human safety and health. When removing pesticides from the environment, it is imperative to ensure high removal efficiency while preventing secondary pollution to the environment. Because of the low concentrations of pesticide residue in the environment, complex matrix, and large throughput of pesticide residue analysis, a low-cost fast pre-treatment technique that has strong selectivity and an enrichment effect on the target pesticide residue, with little environmental impact, is required. Plant biomass-derived biochar is obtained from wheat straw, corn cob, rice husk, etc. This material has a large specific surface area, high pore capacity, tunable surface functional groups, and good environmental compatibility, which make it an inexpensive and efficient adsorbent. Hence, there is a need to systematically review the knowledge regarding the application of plant-based biochar on pesticide removal and pesticide residue analysis. This paper reviews the application progress of plant biomass-derived biochar in the above mentioned two areas over the last decade. The pesticide removal applications include reducing the mobility of pesticides in soil, eliminating the pollution caused by chiral pesticides, loading pesticide-degrading bacteria, and releasing fertilizers sustainably when removing pesticide. As mentioned above, plant biomass-derived biochar has a large specific surface area, a high number of functional groups on the surface, and good environmental compatibility. Therefore, it can effectively remove pesticides or their metabolites from the environment without causing any secondary pollution. During pre-treatment, plan biomass-derived biochar is used as an adsorbent for dispersive solid-phase extraction, solid-phase microextraction, and magnetic solid-phase extraction to selectively adsorb organophosphorus and triazole pesticides in fruits and vegetables, as well as organochlorine pesticides in the aquatic environment. This paper also introduces the adsorption mechanism of plant biomass-derived biochar, where studies based on computational simulations such as the density functional theory, molecular dynamics simulation, and giant canonical Monte Carlo simulation are carefully discussed. The benefits of adopting computational simulations are also mentioned. Finally, this paper summarizes the advantages and disadvantages of using plant biomass-derived biochar in pesticide removal and pre-treatment, as well as the future research trends in this area.

根据欧盟的可再生能源指令,生物质被定义为“来自农业(包括植物源和动物物质)、林业和相关产业(包括渔业和水产养殖业)的生物来源产品、废物和残留物的可生物降解部分,以及工业和城市废物的可生物降解部分”^[1]^。由于减少二氧化碳排放的必要性和化石资源的日益枯竭,生物质作为一种能源和碳源受到了越来越多的关注^[2]^。

图1显示了植物源生物质的主要成分和结构,3种主要成分分别是木质素(27%)、纤维素(43%)和半纤维素(20%)^[3]^。纤维素由数百至数千个葡萄糖单元构成,是植物源细胞壁中的主要物质,其主要作用是保持植物源的坚硬和直立。纤维素有结晶相和非结晶相,它们交织在一起形成微纤维^[4]^。半纤维素由几种不同的五碳糖和六碳糖组成,与纤维素不同,半纤维素是含有大约500~3000个糖单位的较短链^[5]^。木质素由3种酚类构成,分别是对香豆醇、松柏醇和芥子醇,它们分别以对羟基苯基、愈创木基和丁香基的形式结合到木质素中^[6]^。木质素的作用为填充果胶、纤维素和半纤维素之间的细胞壁空间,提供支持植物源结构的机械强度。

**图1:**
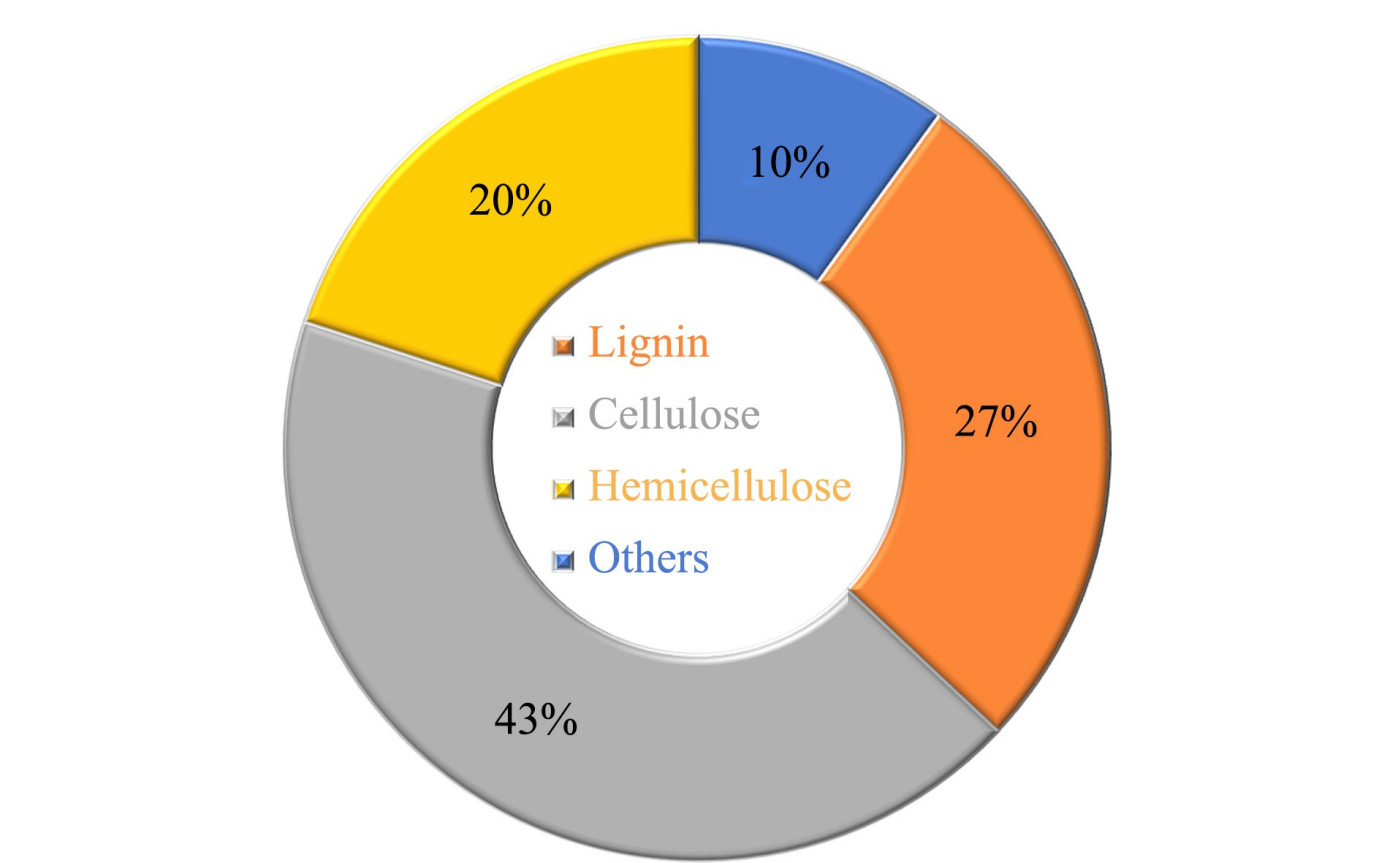
植物源生物质的组成

除了上述3种主要的有机成分,生物质还包括无机成分和其他提取物^[7]^。无机成分包括钾、钠、硅等,其他提取物则包括蛋白质、脂肪和生物碱等。

植物源生物炭材料是指由植物源生物质作为碳源衍生得到的材料,可以采用多种农业废弃物及副产物进行制备,目前常见的作为碳源的生物质有小麦秸秆、玉米芯、稻壳等^[8]^。由于碳源的多样性,植物源生物炭材料的种类多,制备原材料储量丰富,分布广泛。同时,与合成高分子材料等材料相比,植物源生物炭材料的组成使其具有良好的生物降解性,可以在自然环境中很快被微生物降解为水、二氧化碳和小分子,对环境十分友好。

农药是世界上使用最广泛的化学品之一,农药的使用极大地提高了农业生产力,为保护粮食安全做出了重要贡献。然而,农药的过度使用会污染农田,并对环境(水、土壤和空气)和人类的健康造成威胁^[9]^。此外,还有一些农药不易降解,甚至可以远距离的迁移。目前,农药已经普遍存在于环境中,如土壤、空气和水^[10]^。因此对环境中的农药进行监测,并且尽可能去除环境中的农药是对公众健康的保护。

植物源生物炭材料应用于农药领域具有天然的优势。首先,植物源生物质的种类多,分布广,易获得。其次,植物源生物炭材料本身具有良好的物化性质,是一种优秀的吸附材料,同时其拥有可再生和生物降解性良好等优点,应用在农药领域不仅经济性好还能防止材料本身对环境造成污染。本文首先介绍了制备生物质炭材料的常用方法以及为改善其吸附性能而采取的一系列改性方法。文章重点综述了植物源生物炭材料作为吸附剂在农药去除和农药残留分析前处理领域的应用现状,并对其应用的方式和优势进行了讨论。同时,文章也介绍了生物炭材料对于农药分子吸附机理,并综述了目前基于密度泛函理论、分子动力学模拟等理论计算方法探究吸附机理的研究进展,简述了理论计算的优势。最后,本文总结了植物源生物炭材料在农药去除与农药残留分析领域应用的优缺点,指出了尚待解决的问题,并对未来的研究方向做出了展望。

## 1 生物炭材料的制备和改性

生物质作为碳源的好处是成本低,可再生,对环境友好等,目前制备生物炭材料的方法主要有热解、水热碳化和微波辅助碳化,其中热解是目前应用最广泛、最成熟的生物炭材料的制备方法。同时,热解等方法制得的生物炭材料总孔体积小、比表面积小而且表面官能团少,使材料的吸附能较弱。通过一些物理或化学活化方法对生物炭材料进行改性十分必要,这些改性可以大大增强生物炭材料的性能。

### 1.1 生物炭材料的制备

生物质的热解是一个热化学过程,在400~700 ℃的有限供氧或无氧条件下,生物质大部分转化为气体、生物油和生物炭。对于植物源生物质的组成来说,半纤维素在200~250 ℃的温度下首先分解,纤维素在240~350 ℃的温度范围内分解,木质素在280~500 ℃温度下最后分解^[11]^。考虑到生物质的完全炭化,一般热解温度高于500 ℃。

热解温度对生物炭材料的产率、孔径分布和表面化学性质等产生影响。一方面,过高的热解温度会降低生物炭材料的产量^[12]^。另一方面,热解温度升高可以降低产物的平均孔径,提高产物的总孔体积,增大其比表面积^[13]^。热解温度还可以通过影响生物炭材料表面官能团的形成来影响其表面的化学性质,比如含氧官能团在热解温度低时较多,这时得到的生物炭材料大多是亲水性的^[14]^。

水热炭化是一种在水溶液中生产生物炭材料的方法,与热解相比水热炭化温度一般低于350 ℃,是替代热解需要高温热处理的方法^[15]^。水热炭化涉及一系列化学反应,如水解、脱水、脱羧和水存在下的再缩合等^[16]^。采用水热炭化法制备的生物质材料表面具有更多的含氧官能团^[17]^。

水热炭化受温度、反应时间和反应物比例等有关。与热解相同,温度高时会降低生物炭材料的产率,但是会增加材料的总孔体积和比表面积^[18]^。与热解得到的产物相比,水热炭化得到的产物比表面积更低,稳定性更差^[19]^。因此,大部分研究人员更倾向于采用热解的方法得到生物炭材料。

微波辅助技术是缩短热解反应时间、提高不同原料产品质量的有效方法^[20]^。微波辅助炭化的原理是在给样品材料施加高频电压时,具有永久偶极矩的分子排列的方向与外加电场的方向相反,从而通过偶极-偶极旋转产生热量^[21]^。因此微波辅助热解可以更均匀的加热生物质,利用微波在样品内部传播的特点可以节约能源。在微波辅助炭化中,生物炭材料的性能和产率主要受加热速率和辐射功率的影响^[3]^。已有研究证明在微波辅助炭化中加入酸、碱或其他的化学试剂可以提高产物的性能^[22]^。

### 1.2 生物炭材料的改性

物理活化的方法通常使用一些气体如空气、CO_2_或水蒸气等对生物炭材料进行改性^[23]^。使用水蒸气进行物理活化廉价而简单,可增加生物炭材料的比表面积。同时,水蒸气分子中的氧可以与生物炭材料表面的碳反应,有助于产生羧基、羟基等含氧官能团^[24]^。CO_2_也常被用于物理活化,以得到更高碳含量的生物炭材料。此外,CO_2_活化所得的生物炭材料比空气和水蒸气活化得到的含更多微孔和更高的比表面积^[25]^。使用氮气则可以在生物炭材料表面形成氨基等含氮官能团。物理活化对于产物性能的影响主要取决于活化的时间和温度。

化学活化是用化学试剂浸渍生物炭材料进行反应的活化方法。化学活化可以采用酸、碱或盐作为活化剂。这些活化剂可以改善生物炭材料的表面结构并在其表面产生亲水或疏水的官能团以增强其吸附性能。表1罗列了使用酸、碱或盐作为活化剂制备的生物炭材料的性质。

**表1:** 化学活化生物炭材料的性质

Activator	Physical and chemical properties	Ref.
H_3_PO_4_	larger surface area and larger portion of mesoporous	[26]
HNO_3_	increased the cation exchange capacity and number of carboxylic acid groups	[27]
H_2_SO_4_	increase the porosity in carbon structure and number of -SO_3_H groups	[28]
KOH	higher microporosity and the larger specific surface area	[29]
ZnCl_2_	larger specific surface area and mesoporous structure	[30]

除了活化试剂的种类,过量使用活化剂,活化温度过高或活化时间过长都可能使生物炭材料的性能下降。Masoumi等^[31]^采用KOH对藻类生物质进行活化,结果发现,在KOH与藻类的质量比为1.5时能达到较好的产率和总孔隙度,过量的活化剂反而会因为破坏孔,从而降低产物的比表面积。对于活化温度,Qiu等^[32]^使用KOH活化核桃壳炭材料,当温度上升到800 ℃时产物的比表面积增加到1101.4 m^2^/g,再将温度升高到900 ℃时,比表面积反而下降到1033.0 m^2^/g。活化时间的变化也对生物质材料有较大影响,过长的活化时间可能减少总孔体积和平均孔径,这可能是因为孔的塌陷。因此在进行化学活化时,热解前应对活化剂与生物质的比例、温度和时间进行控制,并使之均匀混合。

采用杂原子掺杂的方法可以改善生物炭材料的物理性质(如比表面积)和化学性质(如稳定性)。比如含N掺杂可以增加材料表面极性,促进与目标分子之间的相互作用^[33]^。O掺杂可以通过在生物炭材料表面修饰含氧基团,通过与目标分子形成氢键进而增加材料的吸附能力。S掺杂可以增加材料对重金属的亲和力^[34]^。N、S共掺杂的材料可以提供高催化位点,对氧化还原反应表现出优异的电催化活性^[35]^。

为了提高生物炭材料的吸附和分离能力,将其负载在磁性材料上也已成为一种广泛使用的方法。磁性生物炭材料不仅具有超顺磁性,可以在外磁场的作用下快速分离,同时Fe_3_O_4_可以与污染物之间产生静电作用,有强大的从水溶液中去除重金属的能力^[36]^。Sirajudheen等^[37]^以辣木种子为碳源包裹MnFe_2_O_4_粒子,制备了含碳生物质磁性材料,对水中的3种染料都有较好的吸附能力,同时可以通过磁铁将材料从水中回收,表明该材料是一种廉价并且可以循环使用的吸附剂。Ahmed等^[38]^制备了单独以橙皮制得的生物炭材料,以及采用纳米Fe_3_O_4_颗粒与橙皮共同制得的磁性生物炭材料,对比了两种材料对染料的吸附能力,发现磁性橙皮材料对染料的最大吸附量远高于橙皮材料,且易分离,有良好的可重复使用性。基于以上优点,磁化的各种生物炭材料已被用于农药残留的前处理中。

## 2 植物源生物炭材料在农药领域中的应用

### 2.1 在农药残留去除中的应用

植物源生物炭材料具有高的比表面积、孔隙度以及表面存在大量官能团等优点,可以有效吸附农药或其代谢物,从而显著减少环境中的农药污染,是高效的农药吸附剂(见图2a)^[39]^。Yoon等^[40]^使用葡萄渣制备的生物炭材料可对杀菌剂霜脲氰(CM)进行高效吸附,在pH为7时对霜脲氰的最大吸附量达到了161 mg/g。不同的生物炭原料对农药的吸附效果存在差异,解钧^[41]^通过各种表征手段研究了水稻壳、大豆秸秆、玉米秸秆和玉米芯制备的4种生物炭的结构,发现水稻壳生物炭炭化程度高,抗氧化能力强,具有更发达的孔隙结构,将其应用于田间莠去津、乙氧氟草醚的修复中,经5个月的监测显示,在高浓度处理组中水稻壳生物炭对莠去津、乙氧氟草醚的横向迁移截留率分别可达89%与82%。

**图2:**
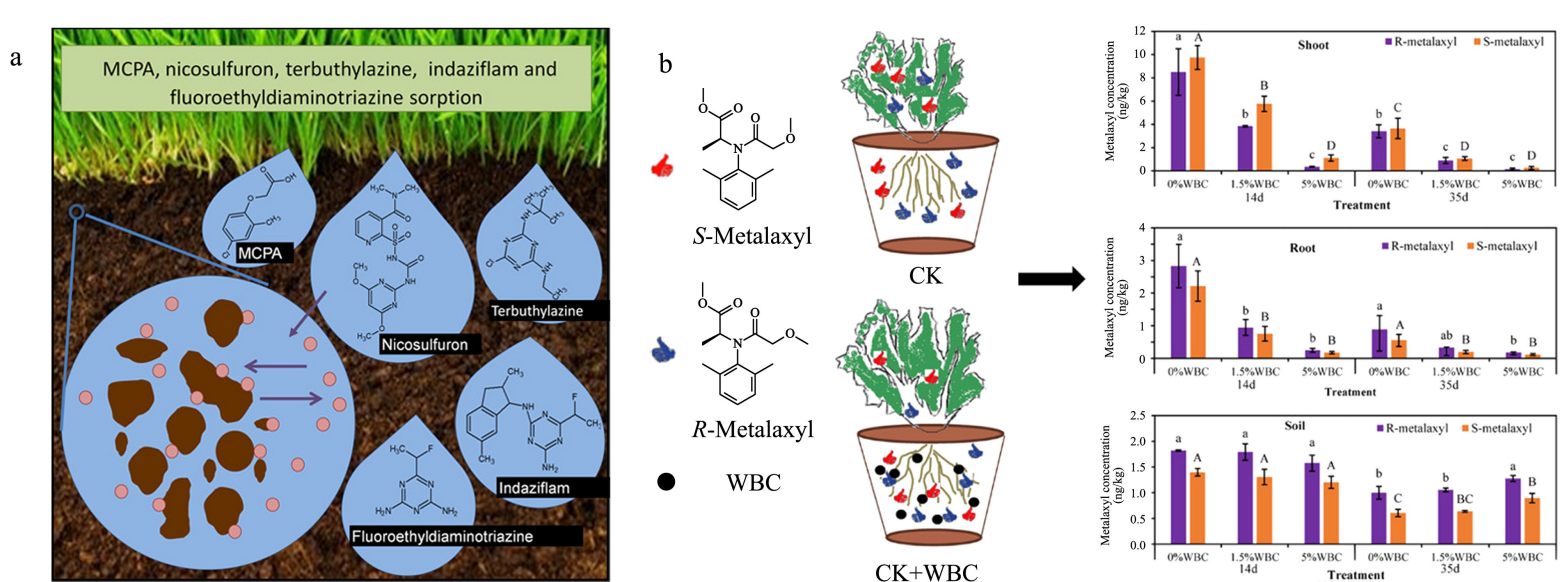
生物炭对土壤中农药的吸附与环境修复作用

不同的改性方法也会影响生物炭对农药的吸附效果,Baharum等^[42]^采用碳化(BC1)、活化(BC2)、磷酸(BC3)、氢氧化钠(BC4)改性制备了椰壳生物炭应用于吸附农药二嗪农,结果表明,在pH值为7、质量浓度为5.0 g/L条件下,BC3是农药二嗪农的高效吸附剂,对二嗪农的去除率高达98.96%,最大吸附容量为10.33 mg/g,其次是BC2(9.65 mg/g)。生物炭对农药的吸附可以降低其在土壤中的淋溶,邢泽炳等^[43]^研究了柠条生物炭吸附敌草隆的土壤淋溶,发现当生物炭含量为3%时能有效吸附敌草隆,6 d时滤出液中敌草隆浓度仅为对照组的36%,柠条生物炭可以有效降低敌草隆向空气中挥发和地下迁移。

植物源生物炭材料还可以对具有手性的农药进行特异性吸收,为手性农药污染的高效去除提供了新途径(见图2b)^[44]^。You等^[45]^通过生菜的盆栽实验,发现木材生物炭(WBC)可以对手性农药甲霜灵(MET)产生特异性吸收并改变土壤细菌群落,从而增加降解细菌的丰度,由此降低了生菜对手性农药甲霜灵及其代谢物的吸收和积累。在Gong等^[46]^的研究中也发现生物炭材料可以特异性吸收手性农药氟虫腈,从而可以用来降低污泥渗滤液中氟虫腈及3种代谢物的污染,同时也观测到生物炭降低了这些污染物对生物体的不利影响。这些实验表明生物炭材料也可用于修复手性农药造成的污染。

在单独使用植物源生物炭材料作为吸附剂时,带负电的生物炭材料与一些带负电的农药比如草甘膦之间的静电排斥会阻碍吸附过程,通过在生物炭材料表面掺杂其他材料可以改善这一问题。Chen等^[47]^通过将对有机磷农药有良好吸附亲和力的金属氧化物纳米颗粒MnFe_2_O_4_负载在纤维活性炭(CAC)上来赋予其正电性能,从而避免了吸附过程的静电排斥,同时此举也可以提高MnFe_2_O_4_的分散稳定性。在使用这种材料吸附农药草甘膦,在pH为4左右时对草甘膦的最大吸附量(167.2 mg/g)远远高于未进行掺杂的CAC(61.44 mg/g)和MnFe_2_O_4_(93.48 mg/g)纳米颗粒。

此外,通过在植物源生物炭材料表面接种可降解农药的细菌也可实现环境中农药的高效去除。植物源生物炭材料的良好吸附性使得菌株可以在其表面牢固的固定或负载,增加了菌株的浓度和寿命,从而提高具有农药降解能力的菌株对于环境中农药的降解效率。Tao等^[48]^在铁改性的植物源生物炭材料上负载了特异性降解莠去津的DNS32菌株(bFeMBC),将其用于环境中莠去津的去除与降解,结果表明菌株的降解效率大大提高,几乎所有的莠去津都被bFeMBC降解,降解率比原始材料提升了11.46%。Wahla等^[49]^通过将4种嗪草酮降解菌组成的细菌联合体MB3R固定在生物炭上使其对土壤中嗪草酮修复潜力得到提高,同时固定在生物炭上的联合体MB3R也修复了嗪草酮对土壤微生物种群和植物生长的有害影响。

植物源生物炭材料在去除环境中农药的同时,也可作为化肥的缓释载体,进一步提高其经济效益。An等^[50]^通过棉花秸秆和H_3_PO_4_共热解开发了载磷生物炭基肥料,其可在对高效氯氟氰菊酯进行高效吸附的同时缓释磷肥,实验表明其对高效氯氟氰菊酯的最大吸附量达到55.90 mg/g,同时负载的磷元素在水中30 d内释放率达到100%。

### 2.2 在农药残留分析中的应用

农药残留分析是食品检测中重要的工作内容,由于农药残留的痕量和基质的复杂性,想要获得可信的检测结果,简化农药残留的检测过程,样品的前处理技术是农药残留分析中的关键环节之一。适当的样品前处理方法可以有效分离和富集目标分析物,降低样品基质及其他杂质的干扰,从而提高分析结果的灵敏度、准确性和可靠性,是当代分析化学重要的研究方向之一^[51]^。目前用于农药残留分析前处理的常用方法主要有固相萃取(SPE)^[52]^、固相微萃取(SPME)^[53]^、分散固相萃取(DSPE)^[54]^、液液萃取( LLE)^[55]^、分散液液微萃取(DLLME)^[56]^等。与固相萃取、固相微萃取和分散固相萃取相比,液液萃取需要大量的溶剂,分散液液微萃取操作则较为复杂,固相萃取、固相微萃取和分散固相萃取成为农药残留分析中应用较多的前处理方法。在固相萃取、分散固相萃取等方法中吸附剂是影响农药提取的关键因素之一,植物源生物炭材料以其比表面积大、孔容量大和表面官能团丰富等性质可以作为农药残留前处理中的新型吸附剂,如图3所示^[57⇓-59]^。

**图3:**
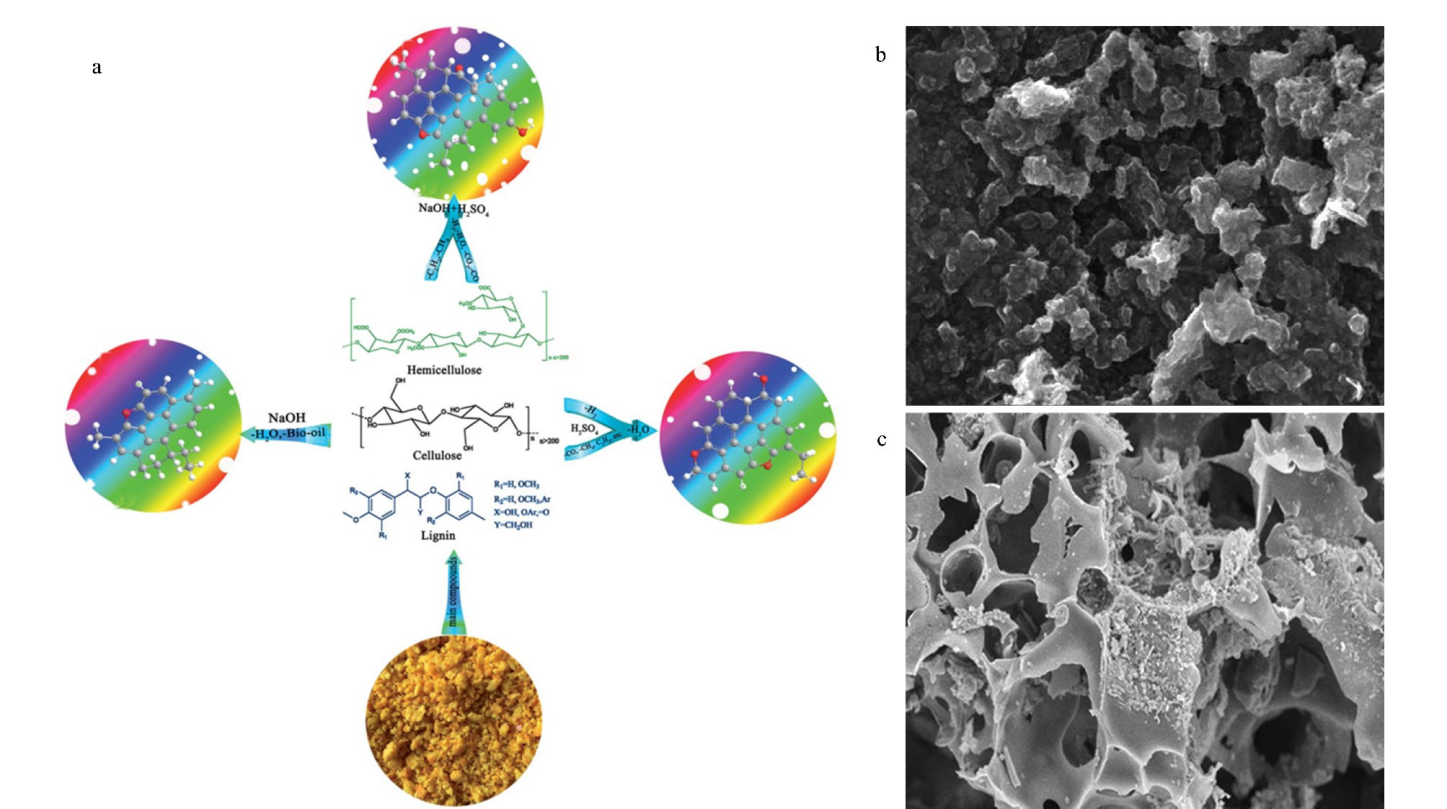
用于样品前处理的生物炭材料

QuEChERS方法是目前农药残留分析中最常用的样品前处理技术之一,其净化部分采用分散固相萃取的方法,常用的净化剂包括*N*-丙基乙二胺(PSA)、石墨化炭黑(GCB)、十八烷基键合硅胶(C18)等材料。近年来制备新型的纳米材料改良QuEChERS已成为当前的研究热点。任科宇等^[60]^采用柑橘皮为原料,ZnCl_2_活化制备了纳米多孔生物炭(NPC),比表面积和孔容分别可达1242 m^2^/g和1.28 cm^3^/g。将NPC用于改良的QuEChERS,测定5种水果蔬菜中14种有机磷农药的检测,与PSA、GCB和C18相比,对不同基质中色素的净化效果NPC>GCB>C18>PSA。在最优条件下,14种有机磷农药检出限为0.63~5.30 μg/kg, 3个添加水平下的平均回收率为71.3%~114.7%,具有极大的实际应用价值。

固相微萃取是在固相萃取和液液分配的基础上开发的一种无需溶剂,集萃取、净化、浓缩为一体的样品前处理技术。萃取涂层的选择是影响固相微萃取的重要因素,涂层的性质决定了整个过程的选择性和灵敏度。Ji等^[61]^以本身含氮的藻类为生物质来源,制备得到了氮掺杂多孔生物炭(NPB), NPB具有发达的孔结构、高含氮量和疏水性,可通过孔隙填充、传质、*π-π*堆积和分配效应促进对氯苯的萃取。采用NPB包覆纤维,结合顶空固相微萃取和气相色谱电子捕获检测器(GC-ECD),建立了一种高效、灵敏的水环境中7种氯苯的检测方法。该方法线性范围宽、检出限和定量限低,精密度高,用于实际水样中氯苯的测定,具有较高的灵敏度和回收率。Behbahan等^[62]^以香蕉皮为碳源,使用热解法制备生物质改性炭材料(BPBs),过程中对活化温度、活化剂、浸渍比和活化次数进行了优化,其中使用H_3_PO_4_作为活化剂使得材料表面具有富氧正电结构,使用ZnCl_2_活化使材料在吸附过程中能够作为路易斯酸与目标物进行作用,制得的改性生物炭材料用傅里叶红外光谱(FT-IR)、扫描电镜(SEM)和能量色散X射线光谱(EDS)进行了表征,并将其应用于移液器尖端固相萃取,在对提取条件进行优化后,结合UHPLC-MS/MS对蔬菜中2,4-二氯苯氧乙酸(2,4-D)、毒死蜱和二嗪磷等12种农药检出限达到0.03~10 μg/L。

经过磁改性的植物源生物炭材料可以在外磁场的作用下快速分离,为吸附物的脱附和材料的重复使用提供了有效途径,使前处理过程的操作更加简便,成本更低。Ren等^[63]^使用废弃柚子皮作为碳源,通过一步水热法合成了包裹Fe_3_O_4_的磁性柚皮生物质材料(C/Fe_3_O_4_ NCs),将其作为萃取剂应用于磁性固相萃取,萃取完成后可在外磁场作用下与溶液快速分离,实验对水果中11种三唑类农药进行了检测,在优化条件下,检出限为0.12~0.55 μg/L,定量限为0.39~1.85 μg/L,最后用于苹果等样品中三唑类农药的分析,回收率为82.1%~109.8%,相对标准偏差小于8.4%。在磁性生物炭材料表面可以进一步修饰其他物质,以提升其物理化学性质。Marsin等^[64]^制备了Fe_3_O_4_和聚吡咯(PPy)改性的油棕纤维活性炭(OPAC)进行磁性固相萃取,采用场发射扫描电子显微镜、振动样品磁强计和傅里叶变换红外光谱对优化后的OPAC-Fe_3_O_4_-PPy复合材料进行了表征。同时利用Box-Behnken设计优化了磁性固相萃取程序的提取时间、解吸时间、盐用量以及pH值,方法对硫丹和狄氏剂的检出限分别达到7.3 ng/L和8.6 ng/L。在该研究中聚吡咯不仅起到增强吸附的作用,同时也可以作为Fe_3_O_4_和OPAC之间的黏合剂。

## 3 植物源生物炭材料的吸附研究

### 3.1 植物源生物炭材料吸附机理

分析和研究吸附机理有助于了解吸附质和吸附剂之间的吸附性能。目前,通过FT-IR、SEM、X射线光电子能谱技术(XPS)、X射线衍射仪技术(XRD)等表征手段研究生物炭材料的吸附机理已得到了广泛应用。如图4所示,目前已知的吸附机理有氢键作用、静电相互作用、*π-π*相互作用和孔隙填充作用等。

**图4:**
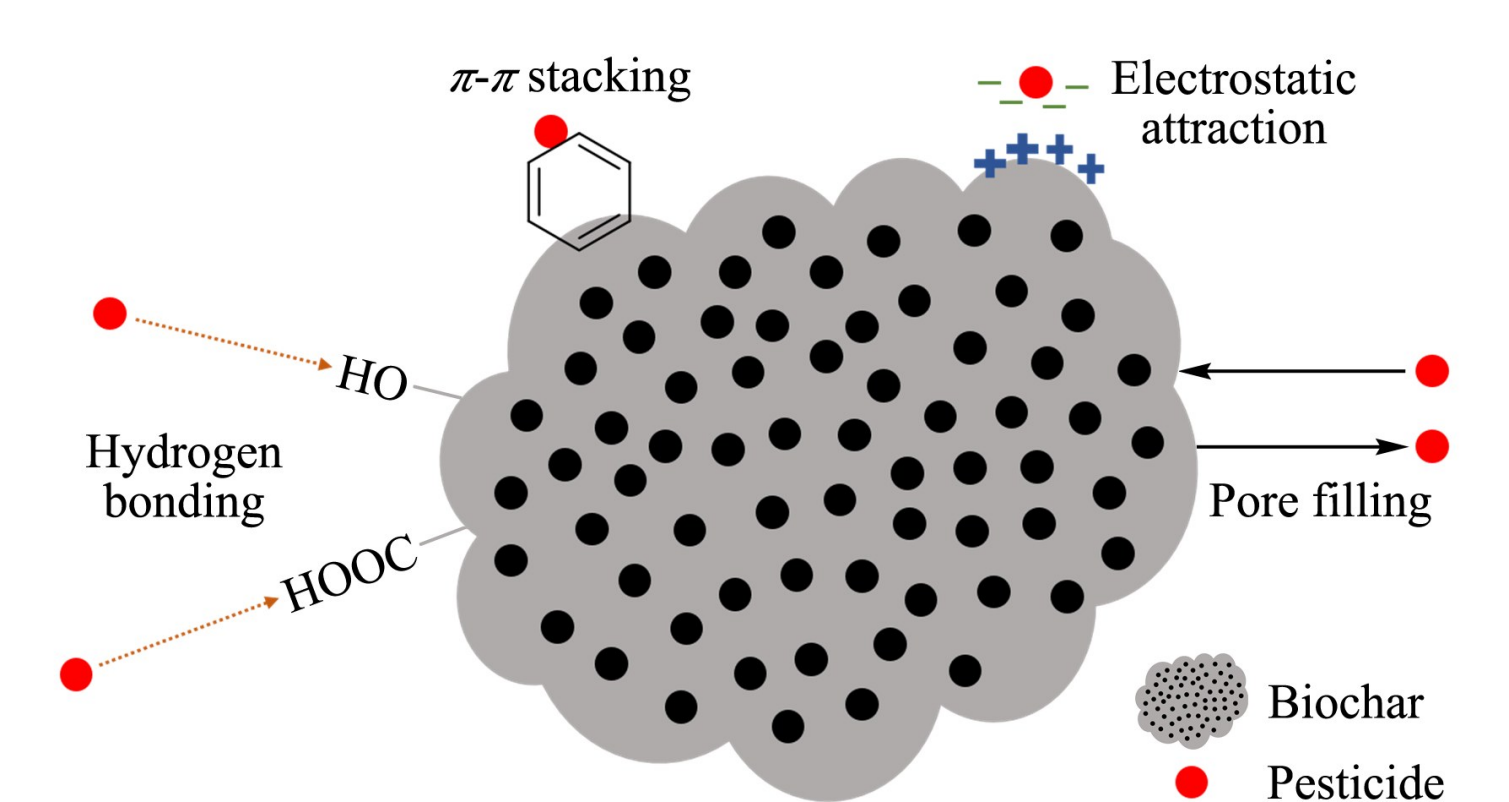
农药在生物炭表面的吸附机理

氢键形成于电负性小的原子(如F、N、O)和功能基团如-OH和-NH的正电性氢核之间^[65]^。由于植物源生物炭材料表面有丰富的官能团,当目标分子存在氢键供体或氢键受体时就会产生氢键作用,从而被吸附。Suo等^[66]^研究了莠去津在生物炭上的吸附机理,发现在FT-IR光谱中,位于1393 cm^-1^的-COOH峰和1224 cm^-1^的P=O峰在吸附前后发生红移同时变宽,说明含氧基团的存在提供了吸附质与吸附剂之间的氢键作用。

静电相互作用是带有电荷的吸附质和吸附剂之间的吸引力。根据农药的 ${{\mathrm{pK}}^{ө}}_{a}$ 值(电离常数的负对数)和植物源生物炭材料表面的等电点的数据,通过调节溶液pH值可以控制农药和生物质材料的静电相互作用。Binh等^[67]^在研究pH值对生物炭对2,4-D的吸附研究中发现,当pH为2.0时2,4-D去除率达到95.0%,而当pH值为4~12时去除率逐渐降低,这是由于2,4-D的 ${{\mathrm{pK}}^{ө}}_{a}$ 值为2.8, pH值为2时2,4-D以带正电的形式存在,同时玉米芯生物炭的零点电荷pH_pzc_为1.1,当pH值大于生物炭的pH_pzc_值时,生物炭的表面电荷为负电荷。因此,在pH值为4~12时,2,4-D的阴离子形式与带负电的生物炭表面之间产生静电排斥,导致2,4-D去除率降低。

*π-π*相互作用是常常发生在芳香环之间的弱相互作用。Binh等^[67]^发现2,4-D拥有的芳香环与材料之间的*π-π*相互作用有助于增强材料对2,4-D的吸附。Tan等^[68]^通过C=C键红外峰值的变化证明了甘蔗渣生物质材料对四环素的主要吸附机理是*π-π*相互作用。

孔隙填充作用与吸附质分子大小以及材料表面的孔径和孔隙度有关,是一种物理吸附。Park等^[69]^在玉米生物炭材料对苯酚吸附特性的研究中发现,随着微孔率的增加,苯酚的吸附量在增加,而表面积与吸附量呈非线性关系,说明材料在对苯酚的吸附过程中存在微孔填充作用。Wang等^[70]^研究发现,脱灰可以增加生物炭的吸附能力,因为脱灰增加了孔隙体积,减小了孔径,从而提供了更多的孔隙填充位置。

### 3.2 基于计算模拟的吸附机理研究

对于吸附质与吸附剂之间的吸附机理研究大部分通过实验方法,根据吸附前后的表征变化结合实验测得的吸附动力学、吸附等温线等数据得出可能的吸附机理。但是这样的方法是从宏观的角度,对于吸附中微观过程很难进行描述。近年来,密度泛函计算、分子动力学模拟和巨正则蒙特卡洛模拟等计算模拟方法已被用于解释吸附质与吸附剂之间的吸附机理,有利于研究人员在分子层面认识吸附过程^[71⇓-73]^。

密度泛函理论是研究物质电子性质的重要方法。20世纪60年代,Hohenberg和Kohn^[74]^提出并证明了密度泛函理论的两大定律,建立起了以电子密度表示能量的密度泛函理论。1965年,Kohn和Sham^[75]^提出了Kohn-Sham方程,介绍了由电子密度计算基态能量的方法。体系总能可表示为:

*E*(*ρ*)=*T*_s_(*ρ*)+*J*(*ρ*)+ ∫ *ρ*( $\overset{-}{r}$ )*V*( $\overset{-}{r}$ )d $\overset{-}{r}$ *+E_xc_*(*ρ*)

其中,*E*(*ρ*)是体系总能,*T*_s_(*ρ*)是体系的动能,*J*(*ρ*)是电子之间的排斥能, ∫ *ρ*( $\overset{-}{r}$ )*V*( $\overset{-}{r}$ )d $\overset{-}{r}$ 是体系中原子核对电子的吸引势,*T_xc_*(*ρ*)是交换相互能。

在巨正则蒙特卡洛模拟中,碳原子被认为是刚性结构粒子,其坐标位置被假定为固定的,碳原子之间的相互作用可以忽略不计^[76]^。吸附剂和吸附质之间的相互作用采用Lennard-Jones势能进行描述:

*U*_LJ_=4*ε_ij_* $\left[{\left(\frac{\sigma_{ij}}{r_{ij}}\right)}^{12}-{\left(\frac{\sigma_{ij}}{r_{ij}}\right)}^{6}\right]$ + $\frac{q_{i}q_{j}}{4\varepsilon_{0}r_{ij}}$

其中,*U*_LJ_表示主客体之间相互作用;*i*和*j*表示不同原子;*r_ij_*表示原子间距;*ε_ij_*和*σ_ij_*为Lennard-Jones参数;*q_i_*和*q_j_*表示原子所带电荷。

目前已经有一些文献报道了理论计算在生物炭材料吸附机理中的研究。Wang等^[77]^结合对比实验和密度泛函计算研究了氮硫双掺杂生物炭对双酚F和双酚S的吸附机理,首先构建了一系列计算模型,通过优化后的几何构型及其相应的电荷密度差图,得到双酚F和双酚S在材料表面的吸附能。最后发现掺杂的氮、硫杂原子起到了协同效应,促进了吸附性能,吸附位点主要是氧化硫和吡啶氮,对于双酚F来说,不同机理的比例贡献遵循疏水作用>*π-π*作用>氢键作用的顺序,而双酚S的吸附机理主要受*π-π*相互作用控制。该工作为双酚的不同性质及其相应的吸附机制提供了新的见解。

两个原子之间的电子云重叠可以反映吸附过程中化学键的形成,并由电荷密度决定。Chen等^[47]^运用密度泛函理论和前线轨道理论模拟分析MnFe_2_O_4_@CAC和草甘膦吸附过程中的电荷密度、前沿轨道和电子态密度。对MnFe_2_O_4_吸附不同价态草甘膦的电子转移、电子云重叠和能隙进行了分析。在吸附过程中电子云重叠结果表明任何价态的草甘膦都能与MnFe_2_O_4_相互作用,形成了新的化学键,并发生电子转移。进一步分析前线轨道发现吸附反应容易自发进行且吸附构型保持高度稳定。最后通过电子态密度分析发现化学吸附过程是由吸附剂的p电子和吸附质的d电子共轭产生的。

Mandal等^[78]^采用分子动力学模拟和巨正则蒙特卡洛模拟预测了磷酸处理的稻草生物炭对莠去津、吡虫啉和嘧菌酯的去除,生物炭的磷酸酯部分在增加生物炭表面的富电子位点中起关键作用,增加了生物炭表面在水介质中的吸附效率。吸附模拟研究还将吸附现象归因于表面-界面相互作用,与多溶质实验结果相吻合。该研究不仅揭示了磷酸处理的稻草生物炭作为一种经济有效的混合污染缓解剂的潜力,而且还为未来量身定制的生物炭表面的预测性设计铺平了道路。

采用理论计算进行植物源生物炭材料吸附机理的研究,其计算结果不仅能对根据实验提出的机理进行支撑,还能预测生物质材料对于目标农药的吸附性能,节约了筛选材料的时间,为材料的选择提供了便利,而且预测结果准确,可信度较高,具有良好的应用前景。

## 4 总结与展望

植物源生物炭材料可以由热解、水热炭化等方法利用废弃生物质制得,并且通过活化、掺杂等方法进行改性,进一步改善其物理化学特性,实现理想的吸附性能。生物质具有原料易得、可再生、环境友好和廉价的优势,其已在农药去除和农药残留分析领域展现出巨大的应用潜力。在农药去除领域,其可作为农药的吸附剂,农药降解菌株的载体和肥料缓释载体。在农药分析领域,生物炭材料具有高的吸附性能,可用做前处理过程中的吸附剂并通过磁改性实现快速分离。同时,理论计算的发展使得如今可以使用计算模拟研究生物质材料的吸附机理,并且快速筛选吸附目标农药合适的植物源生物炭材料。

但是对于植物源生物炭材料在农药领域的应用仍然还有许多问题需要研究和解决:首先,生物炭缺乏特异性,使其对环境中痕量目标物的检测存在难度,可以对其进行特异性改造,比如与其他纳米材料掺杂或制备成分子印迹聚合物。其次,现如今对于吸附机理的研究大部分基于实验测得的吸附等温线等数据进行分析,难以得到吸附过程中的微观信息,未来通过理论计算可以对这方面进行进一步解释。但是通过理论计算预测植物源生物炭材料与农药分子之间的吸附行为时未考虑环境中溶液pH、温度以及其他因素的影响,所以在预测环境中植物源生物炭材料对农药残留去除时需要结合机器学习算法,以提升其预测的准确性。最后,植物源生物炭材料的制备与应用都还停留在实验室阶段,距离在工业生产中的大规模应用还有很长的路要走。
